# An unusual cause of delayed hematoma after carotid endarterectomy: a case report

**DOI:** 10.1186/s12893-019-0601-x

**Published:** 2019-09-18

**Authors:** Yi Zhao, Zhichao Lai, Xiaojun Song, Rong Zeng, Changwei Liu, Xuebin Wang, Rui Zhang, Wei Ye

**Affiliations:** Department of Vascular Surgery, Peking Union Medical College Hospital, Peking Union Medical College and Chinese Academy of Medical Sciences, No.1 Shuaifuyuan, Wangfujing St, Beijing, 100730 China

**Keywords:** Carotid endarterectomy, DIC, Postoperative hematoma, Prostate cancer, Surgical complications

## Abstract

**Background:**

Neck hematoma is a complication of carotid endarterectomy, usually occurring in the comparatively early stage postoperatively.

**Case presentation:**

We described a patient developing life-threatening hemorrhage and non-clotting hematoma at a comparatively later stage after CEA. DIC was diagnosed according to the lab results, and the patient underwent re-operation and was supported with blood products until the coagulopathy was corrected. The patient had a history of prostatic hyperplasia and experienced malaise during the hospitalization. Prostate cancer with bone metastases was diagnosed.

**Conclusions:**

This case report describes a rare underlying cause of hematoma after CEA, which reminds us to pay attention to prostate symptoms or related medical history, especially malignancy, in surgical patients, which may result in severe complications.

## Background

Neck hematoma can be a severe complication of carotid endarterectomy (CEA), usually occurring in the early stage after surgery, and it is commonly associated with technical flaws.

We reported a rare case of postoperative neck hematoma with disordered coagulation profile occurring at late stage after CEA. Prostate cancer with bone metastases was diagnosed as the underlying cause. The aim of this report is to have the knowledge about this rare complication and to be aware of such an emergency.

## Case presentation

A 79-year-old male patient presented with occasionally found severe stenosis (80%) of right internal carotid artery without any symptoms in Dec, 2017. Carotid ultrasound indicated the existence of vulnerable plaque and preoperative MRI demonstrated multiple lacunar infarctual lesions in bilateral cerebrums and an old infarction in left occipital lobe. Apart from right internal carotid artery, transcranial doppler sonography (TCD) suggested multiple sites of stenosis or occlusion in intracranial arteries including left middle cerebral artery and siphon carotid artery. For vertebrobasilar circulation, left vertebral artery was occluded, accompanied with stenosis in right vertebral artery and basilar artery. His remarkable medical history included: well controlled hypertension and hyperlipidemia, re-vascularized coronary and lower limb artery, and untreated prostatic hyperplasia. There were no positive findings in preoperative blood test (platelet 382 × 10^9^/L, serum creatinine 1.1 mg/dL). Coagulation profile was also basically normal, with fibrinogen (3.68 g/L) and D-Dimer (3.66 mg/L FEU) slightly elevated. Aspirin had been administrated for 5 years, till the operation day.

A standard right CEA was performed successfully under general anesthesia. The patient recovered smoothly from anesthesia without need to stay in intensive care unit (ICU). Blood pressure was monitored closely after the surgery, with systolic blood pressure below 130 mmHg. Only 10 ml fluids were drained from the surgical wound during the first 24 h postoperatively, and drainage tube was removed after 24 h. Aspirin alone was re-administered on post-operative day 1.

On post-operative day 2, he complained of low back pain and malaise, followed by mild gum bleeding. A small amount of effused bloody fluids and mild neck hematoma were also observed, followed by bleeding from oral and nasal cavity. Oxygen saturation (SpO_2_) dropped to 90% on room air 2 h later, and neck hematoma enlarged without trachea deviation and wheezing heard. The patient’s symptoms worsened within the next one and a half hours. SpO_2_ could barely be maintained around 90% with nasal cannula at 6 L/min, along with increased gum bleeding and agitation. Then in 3 min, the patient’s SpO_2_ suddenly dropped to 74% with face mask at 8 L/min and the patient progressively lost consciousness, with blood pressure 188/97 mmHg and heart rate 100bmp. Lab tests showed that hemoglobin (10.7 mg/dL) and platelet (163*10^9/L) were decreased compared with preoperative results. Coagulation was disordered, including prolonged prothrombin time (22.0 s) and activated partial thromboplastin time (62.5 s), extremely reduced fibrinogen (< 0.4 g/L), and highly elevated D-Dimer (378.55 mg/L FEU), which indicated the existence of disseminated intravascular coagulation (DIC).

**Fig. 1:**
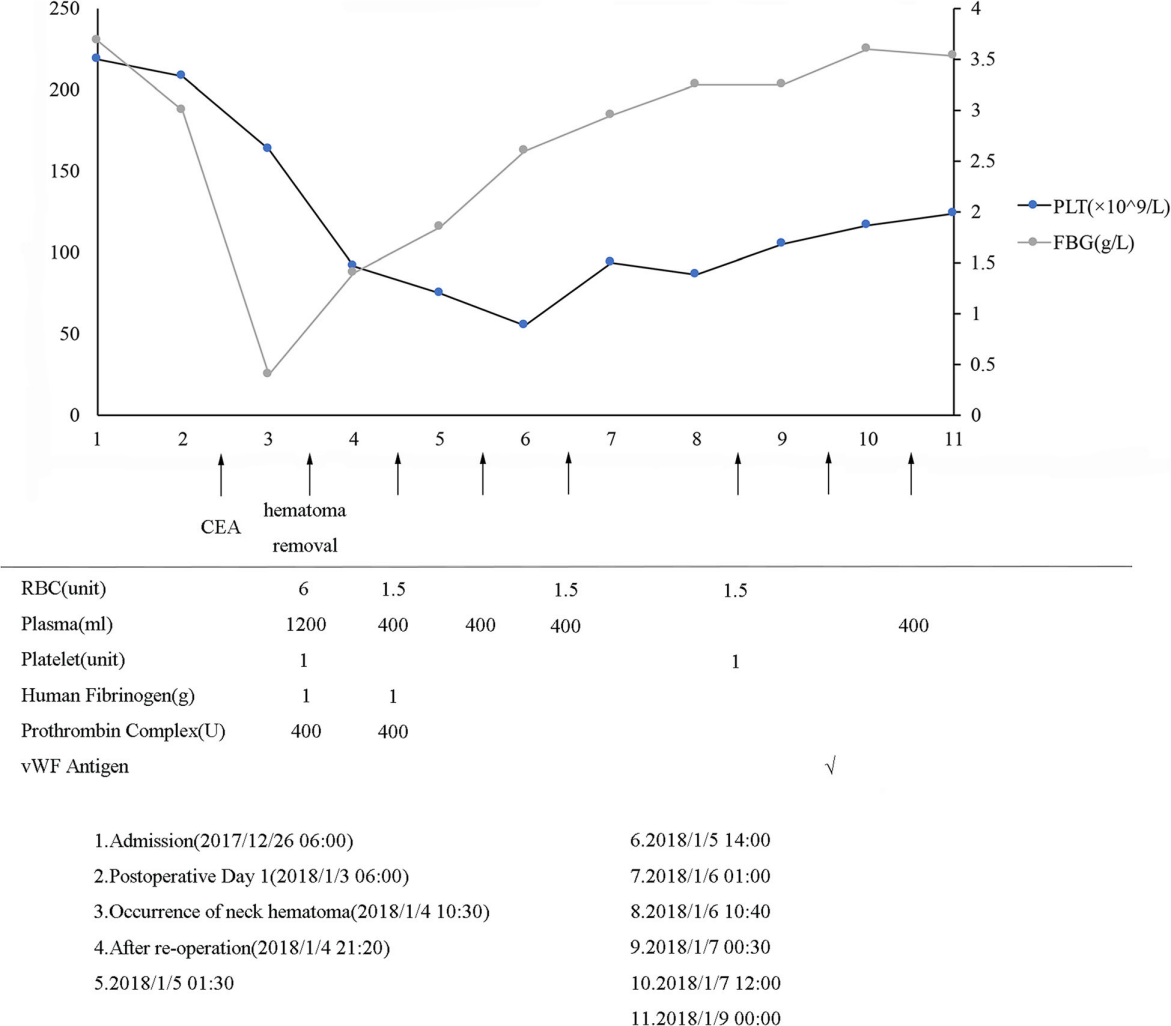
Timeline of interventions (blood products given) and outcomes (platelet)

Emergency rescue was initiated with tracheal intubation and 1 g human fibrinogen administered immediately, and the patient was transferred to operating room for neck hematoma evacuation. A great amount of non-clotting blood was observed inside the wound during the surgery without obvious vessel bleeding and bleeding could not cease spontaneously. Blood products including packed red cells, platelets, fresh frozen plasma (FFP) and prothrombin complex including human fibrinogen and protamine were infused to correct the coagulopathy, as well as hematoma evacuation and pressure hemostasis. The patient’s activated clotting time of whole blood (ACT) went down from 400 s to 180 s at the end of the surgery, indicating that the coagulation function was close to normal and the patient was transferred to ICU. The patient was supported with FFP and platelets in ICU. Over the subsequent 5 days, active bleeding ceased and clotting function was restored gradually. The patient returned to general ward when the condition was stable. In total, 10.5 units of red cells, 2800 ml FFP and 2 units of platelets were administered, as well as 2 g human fibrinogen, 800 U prothrombin complex and vWF antigen. Administration of blood products and the change of platelet are illustrated in Fig. 1.

**Fig. 2:**
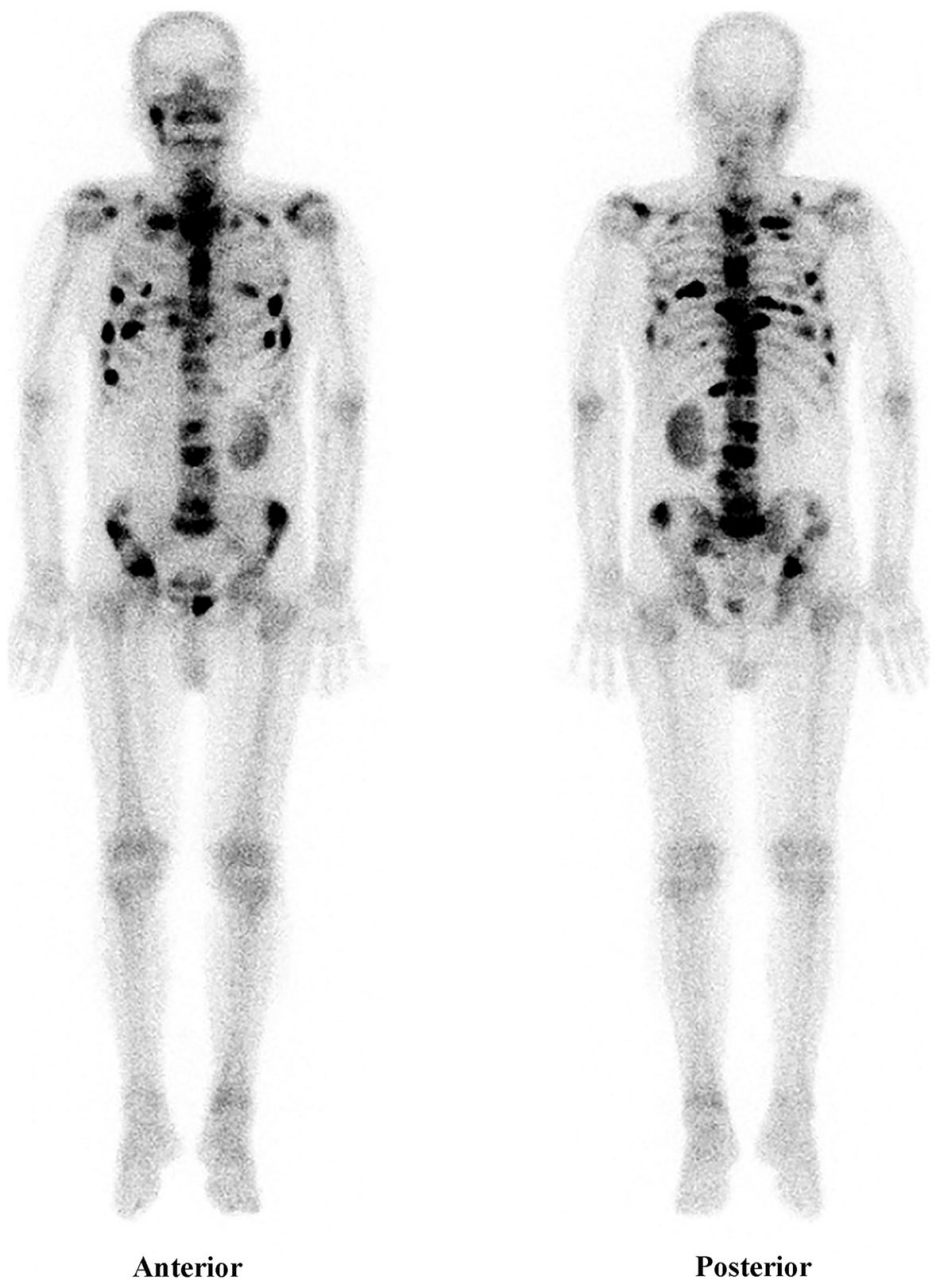
Bone scanning image indicating increased radioactivity uptake in various sites

Once the patient was transferred to general ward, blood test of tumor markers and whole-body bone image were performed to search for potential underlying causes of DIC. Lab analysis revealed elevated level of total prostate-specific antigen (PSA) (1708 ng/ml) and free PSA (> 50 ng/ml). Bone scanning showed multiple areas of abnormally increased radioactivity uptake in right temporomandibular joint, sternum, right clavicle, bilateral ribs and scapulae, as well as spine, pelvis and left proximal femur (Fig. 2), which implied a high probability of bone metastases of prostate cancer. The patient was referred to the Department of Urology. Diagnosis of prostate cancer was confirmed and combination therapy of anti-androgen and gonadotropin-releasing hormone (GnRH) agonist was applied. Anticoagulation therapy (low molecular weight heparin for 1 week, followed by Rivaroxaban for 3 months) were used due to deep venous thrombosis and aspirin were re-administered afterwards. Bleeding, as well as other major complications including stroke and myocardial infarction didn’t occur during a follow-up period of 1 year.

## Discussion and conclusions

Postoperative neck hematoma can be a catastrophic complication of CEA, causing upper airway obstruction, and require surgical intervention, with overall incidence of 3.4% [1]. According to a case-control study, patients who developed neck hematoma had a significantly higher rate of mortality, operative stroke, and myocardial infarction and required more blood transfusions and longer hospital stays [2].

In most cases, neck hematoma developed within 24 h postoperatively, and the mean interval between the completion of CEA and return to operating room for hematoma evacuation was 6 h [3]. In this report, we described a patient developing non-clotting hematoma at a comparatively later stage after CEA. Apart from problems associated with surgical techniques including ligation and electrocoagulation, risk factors for neck hematoma consisted of poor controlled hypertension [4], combined platelet inhibition, use of dextran, and elevated creatinine [2, 5]. Based on medical records and lab test results, the patient didn’t have these risk factors. As the patient was systemically anticoagulated with heparin intraoperatively, combination of non-overt DIC and heparin induced thrombocytopenia (HIT) should be included in differential diagnosis. However, HIT usually occurs in 5–10 days after heparin is administered, much longer than the interval in this case. Thus, diagnosis of DIC was determined due to grossly deranged coagulation profile including low level of fibrinogen.

Massive bleeding can cause DIC, but it is not the case for this patient. First, despite enlarged hematoma, tracheal was central and no wheezing was heard, indicating that the hematoma was not severe enough to compress airway and cause hypoxemia. Aspiration could be a more reasonable explanation for reduced oxygen saturation. Secondly, gum bleeding occurred at the same time of neck hematoma, demonstrating that coagulation disorders were not the result of hematoma, but could be the leading cause of subsequent symptoms. Thus, an underlying cause was sought to explain postoperative DIC, resulting in the diagnosis of prostate cancer with bone metastases.

Theoretically, oncologic patients who undergo surgery are fragile and may have higher risk of peri-operative complications. Solid tumors are reported to be associated with thrombo-hemorrhagic disorder including venous thrombosis, excessive bleeding and evidence of DIC [6]. Patients who have hematologic malignancy can be myelosuppressed and at increased risk of surgery-related infection.

The relationship between prostate cancer and DIC has been described in previously published case reports, and it is put forward that pro-coagulant factors expressing on the surface of tumor cells can trigger the disorder. In most cases, patients are in advanced stage or have metastases when they develop hypofibrinolytic DIC [7], and DIC is also reported to be likely to be the presenting sign of metastatic prostate cancer [8]. Surgery can be a provoking factor, exacerbating the development of DIC [9], which can lead to devastating outcomes. Treatment of the underlying cause as early as possible, namely prostate cancer in this case, is essential to DIC treatment, which can greatly benefit patients. Androgen deprivation including GnRH agonists or antagonists and ketoconazole must be included in the initial treatment to control active bleeding, which can improve prognosis [10]. Thus, although the incidence is rare, it is still meaningful to timely identify high-risk patients. However, as benign prostatic hyperplasia is a common comorbidity in old male patients, which usually co-occurs and shares similar manifestations with prostate cancer including dysuria and increased nocturia, it is difficult to differentiate between these two diseases only through symptoms. It is unrealistic to screen tumors for each patient intending to undergo surgery. Therefore, surgeons should keep this rare complication in mind.

In summary, once unexplained thrombocytopenia or unusual bleeding occurs in patients who have prostatic symptoms during perioperative period, surgeons should be alert to DIC and monitor coagulation profile closely. Furthermore, surgical patients with a history of malignancy should be examined and monitored carefully peri-operatively.

## Data Availability

All patient data and clinical images adopted are contained in the medical files of Peking Union Medical College Hospital in Beijing, China. The data supporting the conclusions of this article are included within the article and its figures. Data sharing is not applicable to this article as no datasets were generated or analyzed during the current study.

## Abbreviations

ACT: Activated clotting time of whole blood
CEA: Carotid endarterectomy
DIC: Disseminated intravascular coagulation
FFP: Fresh frozen plasma
GnRH: Gonadotropin-releasing hormone
HIT: Heparin induced thrombocytopenia
ICU: Intensive care unit
MRI: Magnetic resonance imaging
PSA: Prostate-specific antigen
TCD: Transcranial doppler
